# Bidirectional optogenetic modulation of prefrontal-hippocampal connectivity in pain-related working memory deficits

**DOI:** 10.1038/s41598-019-47555-0

**Published:** 2019-07-29

**Authors:** Helder Cardoso-Cruz, Pedro Paiva, Clara Monteiro, Vasco Galhardo

**Affiliations:** 1 FMUP, Faculdade de Medicina – Departamento de Biomedicina, Universidade do Porto, 4200-450 Porto, Portugal; 2 i3S/IBMC, Instituto de Investigação e Inovação em Saúde e Instituto de Biologia Molecular e Celular - Pain Research Group, Universidade do Porto, 4200-135 Porto, Portugal; 3 FCUP/ICBAS, Faculdade de Ciências e Instituto de Ciências Biomédicas Abel Salazar – Mestrado em Bioquímica, Universidade do Porto, 4169-007 Porto, Portugal

**Keywords:** Working memory, Chronic pain

## Abstract

Dysfunction of the prefrontal-hippocampal circuit has been identified as a leading cause to pain-related working-memory (WM) deficits. However, the underlying mechanisms remain poorly determined. To address this issue, we implanted multichannel arrays of electrodes in the prelimbic cortex (PL-mPFC), and in the dorsal hippocampal CA1 field (dCA1) to record the neural activity during the performance of a delayed non-match to sample (DNMS) task. The prefrontal-hippocampal connectivity was selectively modulated by bidirectional optogenetic inhibition or stimulation of local PL-mPFC glutamatergic calcium/calmodulin-dependent protein kinase-II alpha (CaMKIIα) expressing neurons during the DNMS task delay-period. The within-subject behavioral performance was assessed using a persistent neuropathic pain model – spared nerve injury (SNI). Our results showed that the induction of the neuropathic pain condition affects the interplay between PL-mPFC and dCA1 regions in a frequency-dependent manner, and that occurs particularly across theta oscillations while rats performed the task. In SNI-treated rats, this disruption was reversed by the selective optogenetic inhibition of PL-mPFC CaMKIIα-expressing neurons during the last portion of the delay-period, but without any significant effect on pain responses. Finally, we found that prefrontal-hippocampal theta connectivity is strictly associated with higher performance levels. Together, our findings suggest that PL-mPFC CaMKIIα-expressing neurons could be modulated by painful conditions and their activity may be critical for prefrontal-hippocampal connectivity during WM processing.

## Introduction

The local networks of the prelimbic area of the prefrontal cortex (PL-mPFC) are thought to play a pivotal role in sustaining several cognitive processes in rodents such as working memory (WM)^1–3^. Their function involves the maintenance and processing for a short time interval, of information that is no longer present in the environment and the share of this information to other interconnected brain areas to guide future goal-directed actions^4–7^. Within the PL-mPFC, the maintenance of information is associated with persistent activity of glutamatergic neurons^8–10^, and the altered excitability of these neurons is a central feature of cognitive disorders in a wide range of pathological conditions^11,12^. The timing and spatial selectivity of PL-mPFC glutamatergic neurons are modulated by the inhibitory tonus of local subset of GABAergic interneurons^13–16^, which supports the hypothesis that these local circuitries are important to the regulatory modulation of information and their synchronization share to other brain regions during mnemonic processes.

There is growing evidence that PL-mPFC functioning cannot be separated from that of its densely interconnected areas, such as the hippocampal formation^17,18^. These two brain regions are interconnected by both monosynaptic and polysynaptic interconnections^17^, and there is a strong neural synchrony between them that is regulated by theta oscillations during the behavioral demand^5,7,19,20^. The manipulation of prefrontal-hippocampal circuitries in animals has been associated to cognitive impairments^21–23^. Neonatal hippocampal lesions have a crucial impact in the normal development of prefrontal-hippocampal functional interactions in adult primates, that are necessary for normal performance of WM tasks^24^. Similarly, the prefrontal-hippocampal functional disconnection has been reported in age-related studies^25^, major depression conditions^26,27^, or in patients with neurodegenerative diseases^12,23,27,28^, where there is an increasing evidence that decreased interconnectivity between these two brain regions is associated to a reduction of cognitive performance. In spite of these findings, a circuitry-level understanding of how the prefrontal-hippocampal interplay engages the pain-related WM deficits is lacking.

Recently, we have shown that either neuropathic or inflammatory chronic pain can lead to the manifestation WM deficits in rodents, which are accompanied by a significant disruption in information processing between the prefrontal cortex and other interconnected areas that occurs typically across theta oscillations^29–33^. However, less attention has been paid to the role of local prefrontal excitatory synaptic substrates that contribute to such perturbations during the cognitive demand under pain conditions. Taking into account this question, in this study, we investigated whether the selective bidirectional optogenetic modulation of local PL-mPFC calcium/calmodulin-dependent protein kinase II alpha (CaMKIIα) expressing neurons during the delay-period of a delayed non-match to sample (DNMS) WM task can affect the prefrontal-hippocampal connectivity and behavioral performance in neuropathic pain animals.

## Results

### PL-mPFC CaMKIIα-expressing neurons optogenetic modulation and working-memory task

**Figure 1:**
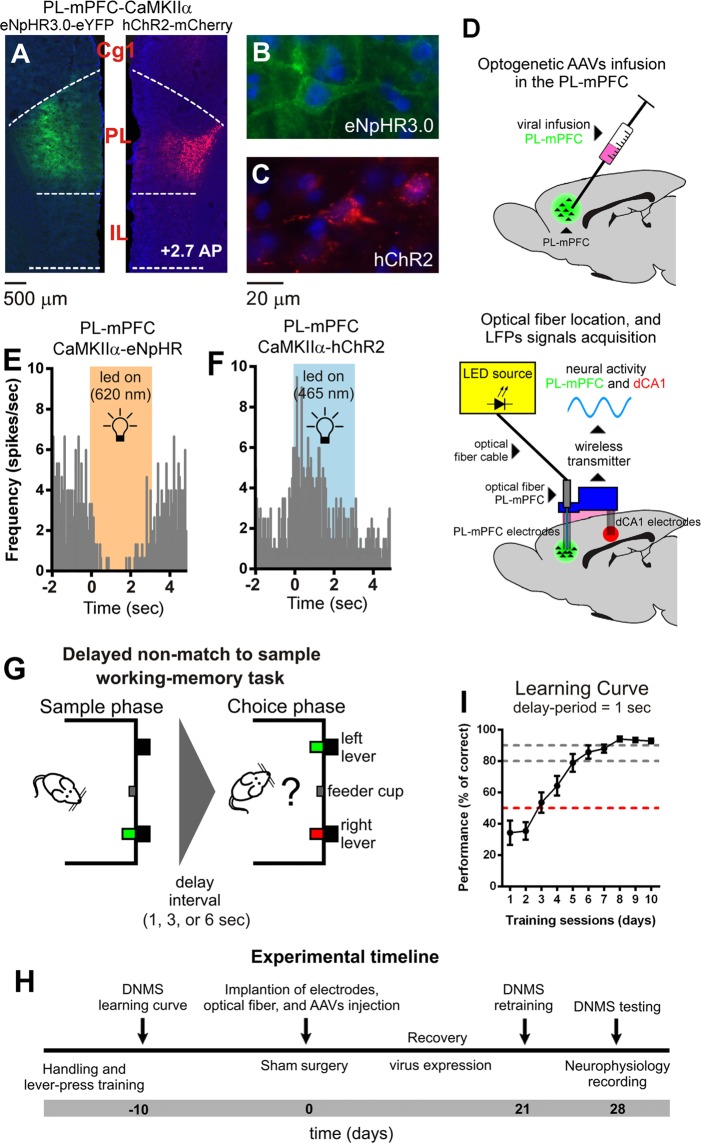
Expression of eNpHR3.0 and hChR2 in PL-mPFC CaMKIIα neurons, neuroelectrophysiological recording locations, functional optogenetic modulation effects in local neural activity, working-memory task and experimental timeline. (**A**) Expression of eNpHR3.0-eYFP (left side) and hChR2-mCherry (right site) in PL-mPFC brain area. Both opsins are expressed in prelimbic (PL), but not in infralimbic (IL) and anterior cingulate cortex (Cg1) regions of medial prefrontal cortex. (**B**) Panel showing an original magnification (x20) of an PL-mPFC eNpHR3.0-expressing neuron, and (**C**) an hChR2-expressing neuron. Blue dots represent the DAPI DNA-labeling. (**D**) The location of optogenetic viral particles infusion and optical fiber in the PL-mPFC, and experimental setup for optogenetic light modulation of local PL-mPFC activity and simultaneous LFPs signals acquisition in the PL and dorsal hippocampus (dCA1). (**E**) Example of the optogenetic inhibition effect in a PL-mPFC CaMKIIα-eNpHR3.0-expressing neuron, and (**F**) example of the optogenetic stimulation effect in a PL-mPFC CaMKIIα-hChR2-expressing neuron. Optogenetic selective inhibition implemented using an orange led light (continuous pulse at 5 mW@620 nm; led on - background orange window), and selective stimulation using a blue led light (pulse duration of 15 ms, frequency of 10 Hz, and a fix intensity of 5 mW@465 nm; led on - background blue window). (**G**) Diagram of delayed nonmatch-to-sample task (DNMS) used in this study. Each trial began with a single lever being exposed (sample phase). When the animal pressed the lever it retracted and the delay-period were initiated. At the end of this period, both levers were exposed (choice-phase) and the animal need to press the opposite lever selected during sample phase to obtain a reward pellet. (**H**) Timeline of the experimental protocol. (**I**) Learning curve, gain in performance during 10 training sessions using 1 s delay-period.

The correct targeting of PL-mPFC area was verified after the behavioral studies by brain cryosectioning. A representative example of extension transfected area in the PL-mPFC is given in Fig. 1A. The right panels showed the magnification of an PL-mPFC CaMKIIα-eNpHR3.0-expressing neuron (Fig. 1B) and a CaMKIIα-hChR2-expressing neuron (Fig. 1C). To verify the physiological effect of illumination in neural activity, we performed extracellular multielectrode recordings in awake rats. In Fig. 1D, we give an illustration of the optogenetic viral infusion and optical fiber location in the PL-mPFC, and experimental setup for light delivery in the PL-mPFC and simultaneous LFPs signals acquisition in the PL-mPFC and hippocampal dCA1. To test the efficiency of viral vector transfection and specificity of light stimulation, we illustrate an example of the changes in discharge activity of a PL-mPFC neuron transfected with AAV-CaMKIIa-eNpHR3.0 (inhibitory opsin, Fig. 1E) and other with AAV-CaMKIIa-hChR2 (excitatory opsin, Fig. 1F), in the absence and during light selective modulation (620 nm orange led light specific to eNpHR3.0 activation, and 465 nm blue led light specific to hChR2 activation). To test the impact of PL-mPFC glutamatergic CaMKIIα-expressing neurons optogenetic manipulation in the behavioral performance, we trained rats in a delayed non-match to sample (Fig. 1G). An experimental timeline of behavioral and surgical procedures is shown in Fig. 1H. Only rats that reached a performance level higher than 90% were subjected to viral injection and optrodes implantation (Fig. 1I).

### Optogenetic modulation did not inhibits neuropathic pain, but induced changes in working-memory performance

**Figure 2:**
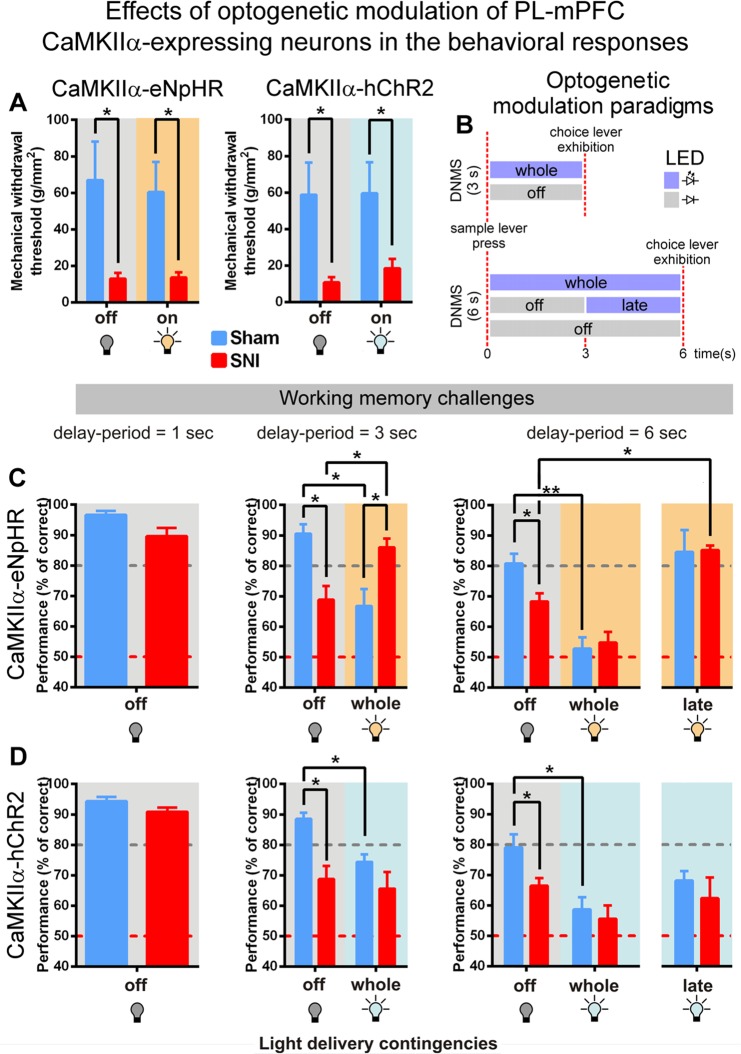
Optogenetic modulation of PL-mPFC CaMKIIα-expressing neurons did not influence pain responses, but induced changes in working-memory performance. (**A**) Effects of contralateral optogenetic inhibition (left panel) and stimulation (right panel) of PL-mPFC CaMKIIα-expressing neurons in mechanical sensivity threshold. (**B**) Illustration of light modulation protocols applied during the delay-period. (**C**) Behavioral performance of eNpHR3.0-expressing rats using a delay-period challenge of 1 (left panel), 3 (middle panel), and 6 s (right panel). (**D**) Behavioral performance of hChR2-expressing rats using a delay-period challenge of 1 (left panel), 3 (middle panel), and 6 s (right panel). eNpHR3.0-expressing rats: sham *n* = 5, and SNI *n* = 5; and hChR2-expressing rats: sham *n* = 5, and SNI *n* = 4. Comparisons between experimental groups and light delivery protocols are based on Mann-Whitney test (for single comparisons) or Kruskal-Wallis test (for multiple comparisons) followed by *post hoc* Dunn’s test. Values are presented as mean ± SEM. *p < 0.05, and **p < 0.01.

To ascertain the role of PL-mPFC glutamatergic CaMKIIα-expressing neurons in pain responses, we assessed mechanical withdrawal thresholds using von Frey filaments. The inhibition of PL-mPFC CaMKIIα-expressing neurons did not reduce pain sensory responses in eNpHR3.0-expressing rats (KW = 15.06, *p* = 0.0018; sham vs. SNI, light off: *p* < 0.05; and light on: *p* < 0.05, Dunn’s *post hoc* test; Fig. 2A, left panel), and the same behavior was observed for the contralateral stimulation protocol in hChR2-expressing rats (KW = 14.00, *p* < 0.0001; sham vs. SNI, light off: *p* < 0.05, and light on: *p* < 0.05; Fig. 2A, right panel).

The performance was tested four weeks after viral infection using different optogenetic modulation protocols applied during the delay-period (Fig. 2B). The effects of contralateral inhibition of PL-mPFC CaMKIIα-expressing neurons in WM performance of eNpHR3.0-expressing rats is presented in Fig. 2C. Data showed no significant differences between experimental groups using 1 s DNMS delay-period challenge in the absence of photomodulation (MW = 13.13, *p* = 0.0952; Fig. 2C, left panel). For 3 s of delay-period (Fig. 2C, middle panel), statistical analysis revealed a significant effect between groups and light delivery protocols (KW = 13.13, *p* = 0.0044); moreover, *post hoc* analysis revealed a higher WM performance for sham-treated rats when compared to SNI-treated rats in the absence of photomodulation (sham vs. SNI, *p* < 0.05; Dunn’s test), and an opposite effect when light modulation was applied to whole delay-period (sham vs. SNI, *p* < 0.05). More interestingly, the inhibition of PL-mPFC CaMKIIα-expressing neurons revealed that SNI-treated rats increased significantly their performance (light off vs. on, *p* < 0.05), whereas the sham-treated rats showed an opposite effect (light off vs. on, *p* < 0.05; Fig. 2C, middle panel). For 6 s of delay-period (Fig. 2C, right panel), statistical analysis revealed a significant effect between groups and light delivery protocols (KW = 21.41, *p* = 0.0007); moreover, *post hoc* analysis shows a higher performance in sham-treated rats when compared to SNI-treated rats in the absence of photomodulation (sham vs. SNI, *p* < 0.05). The inhibition of PL-mPFC CaMKIIα-expressing neurons during the whole delay-period revealed that sham-treated rats decreased significantly their performance (light off vs. on whole delay-period, *p* < 0.01). More interestingly, the photo-inhibition during the late phase of the delay-period reverses the WM impairment of SNI-treated rats (light off vs. late delay-period, *p* < 0.05; Fig. 2C right panel).

The effects of contralateral stimulation of PL-mPFC CaMKIIα-expressing neurons in WM performance of hChR2-expressing rats is presented in Fig. 2D. Data showed no significant differences between experimental groups using 1 s DNMS delay-period challenge in the absence of photomodulation (MW = 4.00, *p* = 0.1905; Fig. 2D, left panel). For 3 s of delay-period (Fig. 2D, middle panel), statistical analysis revealed a significant effect between groups and light protocols (KW = 11.75, *p* = 0.0011); moreover, *post hoc* analysis revealed a higher performance for sham-treated rats when compared to SNI-treated rats without light stimulation (sham vs. SNI, *p* < 0.05; Dunn’s test). In addition, the stimulation of PL-mPFC CaMKIIα-expressing neurons revealed that sham-treated decreased their performance when light stimulation was applied to the entire delay-period (light: off vs. on, *p* < 0.05; Fig. 2D, middle panel). For 6 s of delay-period (Fig. 2D, right panel), statistical analysis revealed a significant effect between experimental groups and light protocols (KW = 11.99, *p* = 0.0349); moreover, *post hoc* analysis shows a higher performance in sham-treated rats when compared to SNI-treated rats in the absence of photomodulation (sham vs. SNI; *p* < 0.05). The stimulation of PL-mPFC CaMKIIα-expressing neurons during the whole delay-period revealed that sham-treated rats decreased significantly their performance (light off vs. on whole delay-period, *p* < 0.05; Fig. 2D, right panel).

### Modulation of PL-mPFC CaMKIIα-expressing neurons induced changes in theta oscillations during working-memory delay-period

**Figure 3:**
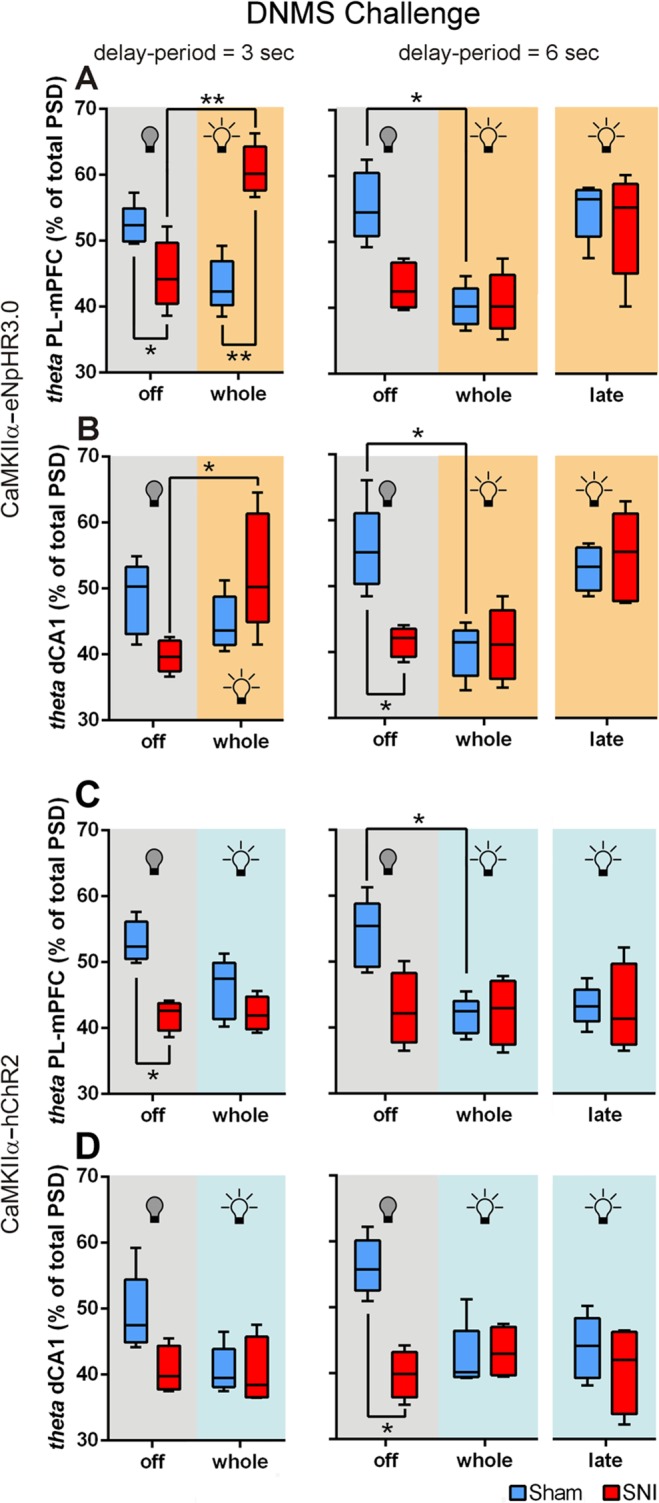
Optogenetic modulation of PL-mPFC CaMKIIα-expressing neurons induced changes in theta power oscillations during working-memory delay-period. Effects of contralateral optogenetic inhibition of eNpHR3.0-expressing rats in (**A**) PL-mPFC and (**B**) dCA1 LFP theta (θ, 4–9 Hz) frequency-band power activity during 3 (left panels) and 6 s (right panels) DNMS delay-period challenges. Effects of contralateral optogenetic stimulation of hChR2-expressing rats in (**C**) PL-mPFC and (**D**) dCA1 LFP theta frequency-band power activity during 3 (left panels) and 6 s (right panels) DNMS delay-period challenges. Data were calculated for each entire recording session independently of correct and incorrect trials. Box-and-whiskers plots are based in the mean and minimum/maximum values. eNpHR3.0-expressing rats: sham *n* = 5, and SNI *n* = 5; and hChR2-expressing rats: sham *n* = 5, and SNI *n* = 4. Comparisons between experimental groups and light delivery protocols are based on Kruskal-Wallis test followed by *post hoc* Dunn’s test. Values are presented as mean ± SEM. *p < 0.05, and **p < 0.01.

The modulation of PL-mPFC CaMKIIα-expressing neurons caused important changes in LFP power oscillations across theta frequency-band during WM delay-period (Fig. 3). In the case of eNpHR3.0-expressing rats, for 3 s of delay-period challenge and PL-mPFC LFPs signals (Fig. 3A, left panel), statistical analysis revealed a significant effect between experimental groups and light delivery protocols (KW = 15.09, *p* = 0.0017); moreover, *post hoc* analysis revealed that sham-treated rats in the absence of light inhibition showed a higher theta activity when compared to SNI-treated rats (sham vs. SNI, *p* < 0.05; Dunn’s test), but this activity was decreased when light inhibition was applied to the whole delay-period (light on whole delay-period: sham vs. SNI, *p* < 0.05). More interestingly, the SNI-treated rats revealed an important increase of their theta activity when light inhibition was applied during the whole delay-period (light off vs. on whole delay-period, *p* < 0.01). In the case of dCA1 LFP signals (Fig. 3B, left panel), statistical analysis revealed a significant effect between groups and light delivery protocols (KW = 9.11, *p* = 0.0279); moreover, *post hoc* analysis revealed that SNI-treated rats increased their dCA1 theta activity when light inhibition was applied during the whole delay-period (light off vs. on, *p* < 0.05). For 6 s of delay-period and PL-mPFC LFP signals (Fig. 3A, right panel), statistical analysis revealed a significant effect between groups and light protocols (KW = 19.49, *p* = 0.0016); moreover, *post hoc* analysis revealed that sham-treated rats decreased significantly their theta activity when PL-mPFC CaMKIIα-expressing neurons light inhibition was applied during the whole delay-period (light off vs. on whole delay-period, *p* < 0.05; Dunn’s test). In the case of dCA1 LFP signals (Fig. 3B, right panel), statistical analysis revealed a significant effect between groups and light protocols (KW = 20.80, *p* = 0.0009); moreover, *post hoc* test revealed that sham-treated rats decreased their theta activity light inhibition was applied during the whole delay-period (light off vs. on whole delay-period, *p* < 0.05). In the absence of light inhibition, sham-treated rats revealed a higher theta activity when compared to SNI-treated rats (sham vs. SNI, *p* < 0.05).

In the hChR2-expressing rats, for 3 s of delay-period and PL-mPFC LFP signals (Fig. 3C, left panel), statistical analysis revealed a significant effect between experimental groups and light protocols (KW = 10.56, *p* = 0.0040); moreover, *post hoc* analysis revealed that sham-treated rats in the absence of light stimulation showed a higher theta activity when compared to SNI-treated rats (sham vs. SNI, *p* < 0.05; Dunn’s test). In the case of dCA1 LFP signals (Fig. 3D, left panel), no statistical differences were observed between experimental groups and light stimulation protocols (KW = 6.99, *p* = 0.0597). For 6 s of delay-period and PL-mPFC LFP signals (Fig. 3C, right panel), statistical analysis revealed a significant effect between groups and light protocols (KW = 11.53, *p* = 0.0494); moreover, *post hoc* test revealed that sham-treated rats decreased significant their theta activity when light stimulation was applied to the whole delay-period (light off vs. on whole delay-period, *p* < 0.05; Dunn’s test). In the case of dCA1 LFP signals (Fig. 3D, right panel), statistical analysis revealed a significant effect between groups and light protocols (KW = 12.60, *p* = 0.0274); moreover, *post hoc* test revealed that sham-treated rats in the absence of light stimulation showed a higher theta activity when compared to SNI-treated rats (sham vs. SNI, *p* < 0.05). The results suggest that higher theta power activity patterns during the WM delay-period are associated typically to higher performance levels.

### Optogenetic modulation induced changes in prefrontal-hippocampal theta coherence and phase-coherence during working-memory delay-period

**Figure 4:**
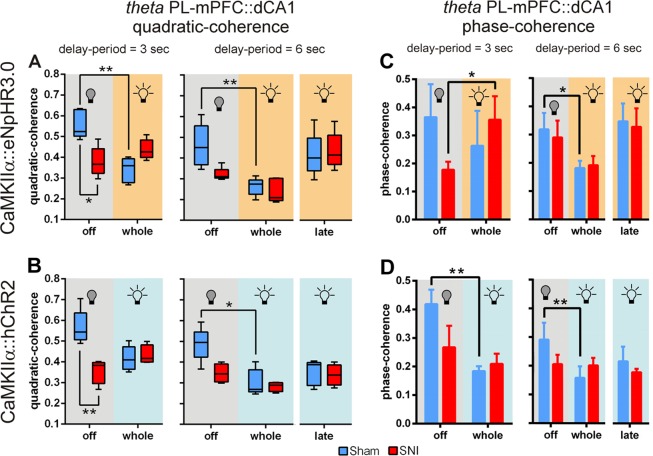
Optogenetic modulation of PL-mPFC CaMKIIα-expressing neurons induced changes in prefrontal-hippocampal theta coherence and phase-coherence during working-memory delay-period. (**A**) Effects of contralateral optogenetic inhibition of eNpHR3.0-expressing rats in prefrontal-hippocampal LFP signals theta (θ, 4–9 Hz) quadratic coherence activity during 3 (left panel) and 6 s (right panel) DNMS delay-period challenges. (**B**) Effects of contralateral optogenetic stimulation of hChR2-expressing rats in prefrontal-hippocampal LFP signals theta quadratic coherence activity during 3 (left panel) and 6 s (right panel) delay-period challenges. (**C**) Effects of contralateral optogenetic inhibition of eNpHR3.0-expressing rats in prefrontal-hippocampal LFP signals theta phase-coherence activity during 3 (left panel) and 6 s (right panel) delay-period challenges. (**D**) Effects of contralateral optogenetic stimulation of hChR2-expressing rats in prefrontal-hippocampal LFP signals theta phase-coherence activity during 3 (left panel) and 6 s (right panel) delay-period challenges. Data were calculated for each entire recording session independently of correct and incorrect trials. Box-and-whiskers plots are based in the mean and minimum/maximum values. eNpHR3.0-expressing rats: sham *n* = 5, and SNI *n* = 5; and hChR2-expressing rats: sham *n* = 5, and SNI *n* = 4. Comparisons between experimental groups and light delivery protocols are based on Kruskal-Wallis test followed by *post hoc* Dunn’s test. Values are presented as mean ± SEM. *p < 0.05, and **p < 0.01.

To determine the spectral coupling among signals of the two recorded areas during the WM delay-period, we calculated the prefrontal-hippocampal LFPs quadratic coherence activity across theta frequency-band. Higher coherence values (near 1) indicate that the signals are identical and totally phase locked at the frequency of interest, whereas a lower value (near 0) indicate that the phases are dispersed. The modulation of PL-mPFC CaMKIIα-expressing neurons caused important changes in prefrontal-hippocampal theta coherence activity (Fig. 4A,B). In the case of eNpHR3.0-expressing rats, for 3 s of delay-period (Fig. 4A, left panel), statistical analysis revealed a significant effect between experimental groups and light delivery protocols (KW = 13.03, *p* = 0.0046); moreover, *post hoc* test revealed that sham-treated rats without light inhibition showed a higher prefrontal-hippocampal theta coherence activity when compared to SNI-treated rats (sham vs. SNI, *p* < 0.05; Dunn’s test). Moreover, the sham-treated rats decreased their prefrontal-hippocampal theta coherence activity when light inhibition was applied to the whole delay-period (light off vs. on whole delay-period, *p* < 0.01). For 6 s of delay-period (Fig. 4A, right panel), we found a significant effect between groups and light protocols (KW = 20.60, *p* = 0.0010); moreover, *post hoc* test revealed that sham-treated rats decreased their prefrontal-hippocampal theta coherence activity when light inhibition was applied to the whole delay-period (light off vs. on whole delay-period, *p* < 0.05). In the hChR2-expressing rats, for 3 s of delay-period (Fig. 4B, left panel), statistical analysis revealed a significant effect between groups and light treatments (KW = 10.75, *p* = 0.0033); moreover, *post hoc* test revealed that sham-treated rats without light stimulation of PL-mPFC CaMKIIα-expressing neurons showed a higher theta coherence activity when compared to SNI-treated rats (sham vs. SNI, *p* < 0.01). For 6 s of delay-period (Fig. 4B, right panel), statistical analysis revealed a significant effect between groups and light treatments (KW = 13.39, *p* = 0.0200); moreover, *post hoc* test revealed that sham-treated rats decreased their prefrontal-hippocampal theta coherence activity when light stimulation was applied to the whole delay-period (light off vs. on whole delay-period, *p* < 0.05).

To determine whether prefrontal-hippocampal phase-coherence in theta frequency-band is modulated by optogenetic manipulations and by the behavioral flexibility required for correct decision-making, we performed phase-coherence analyses of LFP signals obtained from the delay-period activity (Fig. 4CD). In the case of eNpHR3.0-expressing rats (Fig. 4C), statistical analysis revealed a significant effect between experimental groups and light inhibition protocols (3 s of delay-period: KW = 8.40, *p* = 0.0384, left panel; and 6 s of delay-period: KW = 19.84, *p* < 0.0013, right panel). In addition, *post hoc* test used to perform the multiple comparisons revealed that SNI-treated rats increased their theta phase-coherence activity when light inhibition was applied to the whole 3 s delay-period challenge (light off vs. on whole delay-period, *p* < 0.05, left panel; Dunn’s test), and an opposite response in sham-treated rats when light inhibition was applied to the whole 6 s delay-period challenge (light off vs. on whole delay-period, *p* < 0.05, right panel). In the hChR2-expressing rats (Fig. 4D), statistical analysis revealed a significant effect between groups and light stimulation protocols (3 s of delay-period: KW = 10.64, *p* = 0.0037, left panel; and 6 s of delay-period: KW = 15.32, *p* = 0.0091, right panel); moreover, *post hoc* analysis revealed that sham-treated rats decreased their prefrontal-hippocampal theta phase-coherence activity when light stimulation of PL-mPFC CaMKIIα-expressing neurons was applied to the whole delay-period (light off vs. on whole delay-period: 3 s and 6 s, both *p* < 0.05; left and right panels, respectively). Taken together, the results suggest that higher prefrontal-hippocampal theta coherence and phase-coherence values during the WM delay-period are associated typically to higher performance levels.

### Modulation of PL-mPFC CaMKIIα-expressing neurons induced changes in prefrontal-hippocampal connectivity during working-memory delay-period

**Figure 5:**
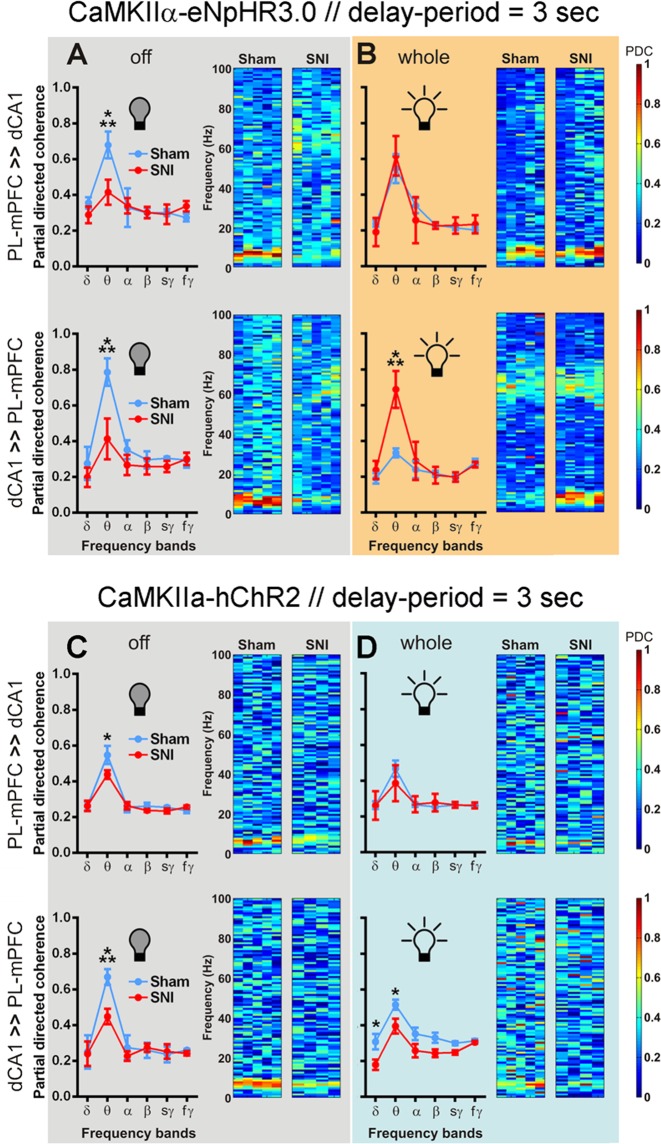
The optogenetic inhibition of PL-mPFC CaMKIIα-expressing neurons alters significantly the prefrontal-hippocampal connectivity in SNI-treated rats during the 3 s delay-period challenge. The bidirectional analysis of prefrontal-hippocampal connectivity (PDC activity) showed that changes in circuitry connectivity occurred mainly at the theta (θ, 4–9 Hz) frequency-band during DNMS task 3 s delay-period challenge. The selective optogenetic inhibition of PL-mPFC CaMKIIα-eNpHR3.0-expressing neurons increased the prefrontal-hippocampal theta connectivity in SNI-treated rats, and decreased from the hippocampus to the prefrontal cortex in sham-treats rats. In the case of activation of PL-mPFC CaMKIIα-hChR2-expressing neurons, both experimental groups shared a decreased of their prefrontal-hippocampal connectivity. The effects of optogenetic inhibition of eNpHR3.0-expressing rats in PDC activity: (**A**) without inhibition (led off); and (**B**) with light inhibition applied during the whole delay-period. The effects of optogenetic activation of hChR2-expressing rats in PDC activity: (**C**) without activation (led off); and (**D**) with light activation applied during the whole delay-period. Top panels indicate PDC activity from PL-mPFC to dCA1 direction, whereas bottom panels indicate PDC activity from dCA1 to PL-mPFC. The respective right panels indicate PDC activity from 1–100 Hz (step resolution of 1 Hz) for each experimental groups and animal. Data were calculated for each entire recording session independently of correct and incorrect trials. eNpHR3.0-expressing rats: sham *n* = 5, and SNI *n* = 5; and hChR2-expressing rats: sham *n* = 5, and SNI *n* = 4. Comparisons between experimental groups across light delivery protocols are based on two-way ANOVA (F1: experimental groups X F2: frequency-bands) test followed by *post hoc* Bonferroni test. Values are presented as mean ± SEM. *p < 0.05, and ***p < 0.001.

**Figure 6:**
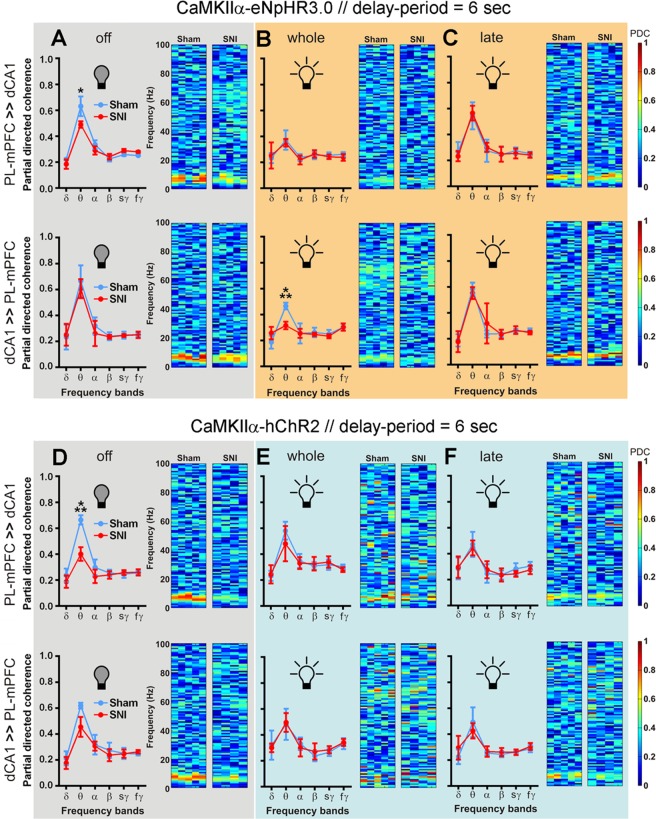
The optogenetic inhibition of the PL-mPFC CaMKIIα-expressing neurons during the late phase of the 6 s delay-period challenge increased the prefrontal-hippocampal connectivity in both experimental groups. The optogenetic inhibition of PL-mPFC CaMKIIα-eNpHR3.0-expressing neurons decreased the prefrontal-hippocampal theta connectivity in both experimental groups during the whole delay-period, but not when applied during the late portion of the delay-period. In the case of activation of PL-mPFC CaMKIIα-hChR2-expressing neurons, both experimental groups shared no significant differences in their prefrontal-hippocampal connectivity (both neuromodulation protocols). The effects of optogenetic inhibition of eNpHR3.0-expressing rats in PDC activity: (**A**) without inhibition (led off); (**B**) with light inhibition applied during the whole delay-period; and (**C**) with light inhibition during the late phase of the delay-period. The effects of optogenetic activation of hChR2-expressing rats in PDC activity: (**D**) without activation (led off); (**E**) with light activation applied during the whole delay-period; and (**F**) with light activation during the late phase of the delay-period. Top panels indicate PDC activity from PL-mPFC to dCA1 direction, whereas bottom panels indicate PDC activity from dCA1 to PL-mPFC. eNpHR3.0-expressing rats: sham *n* = 5, and SNI *n* = 5; and hChR2-expressing rats: sham *n* = 5, and SNI *n* = 4. Comparisons between experimental groups across light delivery protocols are based on two-way ANOVA (F1: experimental groups X F2: frequency-bands) test followed by *post hoc* Bonferroni test. Values are presented as mean ± SEM. *p < 0.05, and ***p < 0.001.

To further study the frequency-dependent relationship between prelimbic and dorsal hippocampus regions during cognitive demand, we estimated the level of functional connectivity by PDC analysis^34^ (Figs 5, 6). The PDC technique is an alternative representation of multivariate processes involving Granger causality models that allows the study of interactions between brain areas revealing their directionality. Higher values of PDC (near 1) indicate the existence of a strong connectivity, and that can be interpreted as the existence of information flow between these structures. It is important to refer that the majority of connectivity oscillations during the delay-period were mediated by changes across theta frequency-band. In fact, when carefully analyzing the connectivity oscillations across this frequency-band in both circuit directions, we can assume that better WM performance levels are associated to higher theta connectivity level. An example of this observation can be found for sham-treated rats without light modulation when compared to SNI-treated rats.

The effects of optogenetic modulation of PL-mPFC CaMKIIα-expressing neurons in the bidirectional prefrontal-hippocampal connectivity oscillations for 3 s of delay-period challenge are illustrated in Fig. 5. In the case of eNpHR3.0-expressing rats and without light modulation (Fig. 5A), ANOVA revealed a significant effect across experimental groups (PL-mPFC ≫ dCA1: F_(1,48)_ = 10.49, *p* = 0.0022, Fig. 5A top panel; and dCA1 ≫ PL-mPFC: F_(1,48)_ = 42.22, *p* < 0.0001, Fig. 5A bottom panel), and across frequency-bands (PL-mPFC ≫ dCA1: F_(5,48)_ = 33.49, *p* < 0.0001, Fig. 5A top panel; and dCA1 ≫ PL-mPFC: F_(5,48)_ = 47.11, *p* < 0.0001, Fig. 5A bottom panel). More importantly, the *post hoc* test revealed that sham-treated rats exhibited a higher theta connectivity when compared to SNI-treated rats (both circuit directions, *p* < 0.001; Bonferroni test). When the inhibition protocol was applied to whole delay-period (Fig. 5B, top panel), from PL-mPFC to dCA1 ANOVA revealed no significant differences between experimental groups, and a significant effect across frequency-bands (F_(5,48)_ = 28.82, *p* < 0.0001). In the opposite direction, from dCA1 to PL-mPFC (Fig. 5B, bottom panel), ANOVA revealed a significant effect between groups (F_(1,48)_ = 28.79, *p* < 0.0001), as well across frequency-bands (F_(5,48)_ = 51.46, *p* < 0.0001); moreover, *post hoc* analyses revealed that SNI-treated rats showed a higher theta connectivity when compared to control rats (*p* < 0.001). In the case of hChR2-expressing rats and without light modulation, from PL-mPFC to dCA1 (Fig. 5C, top panel), ANOVA revealed no significant effect between groups, and a significant effect across frequency-bands (F_(5,42)_ = 30.42, *p* < 0.0001). In the opposite direction, from dCA1 to PL-mPFC (Fig. 5C bottom panel), ANOVA revealed a significant effect between groups (F_(1,42)_ = 10.39, *p* = 0.0025) and frequency-bands (F_(5,42)_ = 59.45, *p* < 0.0001). For both circuit directions the sham-treated rats showed a higher theta activity when compared to SNI-treated rats (*p* < 0.05 and *p* < 0.001, respectively; Bonferroni test). When the optogenetic stimulation of PL-mPFC CaMKIIα-expressing neurons was performed during whole delay-period, from PL-mPFC to dCA1 (Fig. 5D top panel), ANOVA revealed no significant differences between groups, and a significant effect across frequency-bands (F_(5,42)_ = 14.49, *p* < 0.0001). In the opposite circuit direction (Fig. 5D bottom panel), data revealed a significant effect between experimental groups (F_(1,42)_ = 31.01, *p* < 0.0001), and frequency-bands (F_(5,42)_ = 12.64, *p* < 0.0001). In this case, sham-treated rats showed a higher delta and theta activity when compared to SNI-treated rats (both *p* < 0.05).

The effects of optogenetic modulation of PL-mPFC CaMKIIα-expressing neurons in the bidirectional prefrontal-hippocampal connectivity oscillations for 6 s of delay-period challenge are illustrated in Fig. 6. In the case of eNpHR3.0-expressing rats and without light modulation (Fig. 6A), ANOVA revealed no significant effect between groups in both circuit directions, and a significant effect across frequency-bands (PL-mPFC ≫ dCA1: F_(5,48)_ = 35.84, *p* < 0.0001, top panel; and dCA1 ≫ PL-mPFC: F_(5,48)_ = 56.90, *p* < 0.0001, bottom panel). Moreover, *post hoc* test revealed that sham-treated rats showed a higher theta connectivity when compared to SNI-treated rats from PL-mPFC to dCA1 (*p* < 0.05). When the optogenetic inhibition of PL-mPFC CaMKIIα-expressing neurons was performed during whole delay-period (Fig. 6B), in both circuit directions, data revealed no significant differences between experimental groups, and a significant effect across frequency-bands (PL-mPFC ≫ dCA1: F_(5,48)_ = 11.91, *p* < 0.0001, Fig. 6B top panel; and dCA1 ≫ PL-mPFC: F_(5,48)_ = 25.13, *p* < 0.0001, Fig. 6B bottom panel). Interestingly, from dCA1 to PL-mPFC, for this delay-period protocol the sham-treated rats showed a higher theta connectivity when compared to SNI-treated rats (*p* < 0.001). When the optogenetic inhibition was applied during the last portion of 6 s delay-period (late protocol, Fig. 6C), in both circuit directions, data revealed no significant differences between experimental groups, and a significant effect across frequency-bands (PL-mPFC ≫ dCA1: F_(5,48)_ = 45.57, *p* < 0.0001, top panel; and dCA1 ≫ PL-mPFC: F_(5,48)_ = 53.84, *p* < 0.0001, bottom panel). In the case of hChR2-expressing rats and without light modulation (Fig. 6D), ANOVA revealed a significant effect between groups (PL-mPFC ≫ dCA1: F_(1,42)_ = 39.12, *p* < 0.0001, top panel; and dCA1 ≫ PL-mPFC: F_(1,42)_ = 11.04, *p* = 0.0027, bottom panel), and frequency-bands (PL-mPFC ≫ dCA1: F_(5,42)_ = 89.82, *p* < 0.0001, bottom panel; and dCA1 ≫ PL-mPFC: F_(5,42)_ = 65.01, *p* < 0.0001, bottom panel). Moreover, *post hoc* test revealed that sham-treated rats showed a higher theta activity when compared to SNI-treated rats from PL-mPFC ≫ dCA1 (*p* < 0.001). When the optogenetic stimulation was applied during the entire and the last portion of 6 s delay-period challenge (Fig. 6E,F, respectively), data revealed no significant differences between experimental groups, and as expected a significant effect across frequency-bands (PL-mPFC ≫ dCA1: whole delay-period F_(5,42)_ = 27.95, *p* < 0.0001, Fig. 6E top panel; and late delay-period F_(5,42)_ = 14.19, *p* < 0.0001, Fig. 6F top panel; and dCA1 ≫ PL-mPFC: whole delay-period F_(5,42)_ = 16.61, *p* < 0.0001, Fig. 6E bottom panel; and late delay-period F_(5,42)_ = 18.98, *p* < 0.0001, Fig. 6F bottom panel; respectively). Taken together, the results suggest that typically the sham-treated rats exhibit higher prefrontal-hippocampal connectivity levels across the theta frequency-band when compared to SNI-treated rats, but the select inhibition of eNpHR3.0-expressing neurons in SNI-treated rats can decrease these difference.

### Prefrontal-hippocampal theta connectivity can predict the occurrence of correct or incorrect trials

**Figure 7:**
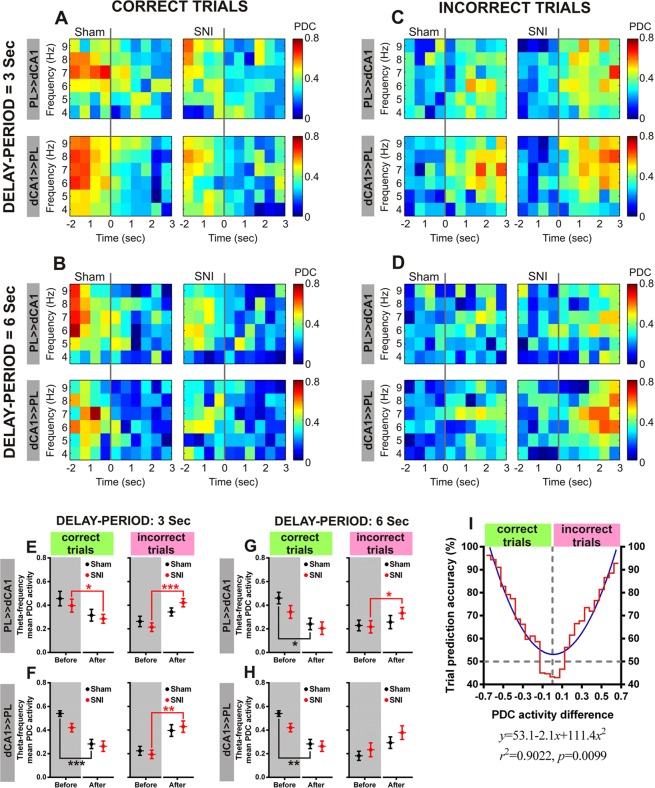
Prefrontal-hippocampal theta connectivity is a good predictor for correct working-memory performance. Perievent raster plots representing the prefrontal-hippocampal theta (θ, 4–9 Hz) connectivity during correct (**A**) 3 s DNMS delay-period; and (**B**) 6 s DNMS delay-period challenge) and incorrect trials (**C**) 3 s DNMS delay-period; and (**D**) 6 s DNMS delay-period challenge). Correct trials are characterized by a transient bidirectional prefrontal-hippocampal connectivity enhancement at theta frequency-band before level press, which decreased after lever press. Incorrect trials are characterized by an opposite response. Each left panel indicate the response to sham-treated rats, whereas each right panel indicate the response to SNI-treated rats. Top panels indicate PDC activity from PL-mPFC to dCA1, whereas bottom panels from dCA1 to PL-mPFC. Frequency-time function computed with a resolution of 1 Hz, and centered at free-choice lever press (time = 0 s; vertical gray line). The averaged bidirectional prefrontal-hippocampal theta connectivity, before and after free-choice lever press, for 3 (**E**) from PL-mPFC to dCA1; and (**F**) from dCA1 to PL-mPFC) and 6 s (**G**) from PL-mPFC to dCA1; and (**H**) from dCA1 to PL-mPFC) DNMS delay-period challenges. Left panels indicate correct trials, and right panels indicate incorrect trials. (**I**) Fitting activity prediction model accuracy behavior. Model computed using randomly 200 trials. This model was used to classify performed trials according to PDC activity oscillation after free-choice lever press. If *PDC*_*d*_ < 0 as a correct trial, otherwise, if *PDC*_*d*_≥0 as an incorrect trial. Comparisons between experimental groups and lever press responses are based on Kruskal-Wallis test followed by *post hoc* Dunn’s test. Values are presented as mean ± SEM. *p < 0.05, **p < 0.01, and ***p < 0.001.

To determine whether prefrontal-hippocampal theta connectivity is related to correct performance, we focused on the PDC activity around the DNMS free-choice lever press (Fig. 7). The global qualitative analysis showed that the correct trials are typically characterized by a transient prefrontal-hippocampal theta connectivity elevation just before the lever press, which occurs in both circuit directions, and by a decrease after free-choice lever press (3 s DNMS delay-period challenge, Fig. 7A; and 6 s DNMS delay-period challenge, Fig. 7B). In the case of incorrect trials, our data showed an opposite response (3 s DNMS delay-period challenge, Fig. 7C; and 6 s DNMS delay-period challenge, Fig. 7D). To further quantify these responses, we computed the mean bidirectional prefrontal-hippocampal theta connectivity, before and after DNMS free-choice lever press across 3 s (PL-mPFC ≫ dCA1, Fig. 7E; and dCA1 ≫ PL-mPFC, Fig. 7F) and 6 s (PL-mPFC ≫ dCA1, Fig. 7G; and dCA1 ≫ PL-mPFC, Fig. 7H) delay-period challenges and in function of correct and incorrect trials (right and left panels, respectively). In the case of 3 s delay-period challenge, from PL-mPFC to dCA1 (Fig. 7E), statistical analysis revealed a significant effect between experimental groups and periods surrounding the free-choice lever press (correct trials: KW = 11.65, *p* = 0.0087 (left panel); and incorrect trials: KW = 18.50, *p* = 0.0003 (right panel)); moreover, *post hoc* analysis revealed that SNI-treated rats during correct trials decreased their theta connectivity after lever-press (*p* < 0.05; Dunn’s test), and increased their theta connectivity during incorrect trials (*p* < 0.001). From dCA1 to PL-mPFC (Fig. 7F), statistical analysis revealed a significant effect between groups and free-choice lever press response (correct: KW = 18.66, *p* = 0.0003 (left panel); and incorrect: KW = 16.01, *p* = 0.0011 (right panel)). Moreover, *post hoc* test showed that sham-treated rats decreased their theta connectivity after perform a correct trial (*p* < 0.001), and SNI-treated rats increased their theta connectivity after perform an incorrect trial (*p* < 0.01). In the case of 6 s delay-period, from PL-mPFC to dCA1 (Fig. 7G), statistical differences were observed between groups and free-choice lever press response (correct: KW = 14.02, *p* = 0.0029; and incorrect: KW = 8.83, *p* = 0.0316). Moreover, *post hoc* test showed that sham-treated rats decreased their theta connectivity after perform a correct trial, and SNI-treated rats increased their theta connectivity after perform an incorrect trial (both, *p* < 0.05). From dCA1 to PL-mPFC (Fig. 7H), we found statistical differences between groups and lever press responses (correct: KW = 14.65, *p* = 0.0021; incorrect: KW = 12.35, *p* = 0.0063). Additionally, in the case of correct trials, *post hoc* test revealed a decreased theta connectivity in the sham-treated animals (*p* < 0.01). In addition, we have used a non-linear polynomial second order function to model the behavioral response based on the prefrontal-hippocampal theta connectivity during free-choice lever press. The accuracy of the computed model in the trial response prediction is illustrated in Fig. 7I. Higher PDC activity differences indicate a strong probability to identify a correct or incorrect trial occurrence, whereas PDC oscillations near 0 decrease significantly the model accuracy.

## Discussion

It is widely assumed that pain interferes with cognitive processing, reducing performance on complex tasks. This assumption suggests that pain processing and cognitive functions engage an overlapping set of capacity-limited general domain brain resources^35,36^. The results of this study show that the induction of a neuropathic pain condition affects performance in a WM task and disrupts prefrontal-hippocampal activity in a frequency-dependent manner. Here, we report that the optogenetic selective inhibition of PL-mPFC local CaMKIIα-eNpHR3.0-expressing neurons during the WM task delay-period reduces pain-related cognitive deficits, and enhances prefrontal-hippocampal theta frequency-band connectivity during behavioral performance. In contrast, activation of PL-mPFC local CaMKIIα-hChR2-expressing neurons induces a significant reduction of WM performance. Finally, we found that prefrontal-hippocampal theta connectivity is strictly associated to higher performance levels, and their oscillation analysis can be used to predict the success of future responses.

At the behavioral level, our findings suggest that the selective optogenetic inhibition of CaMKIIα-eNpHR3.0-expressing neurons is able to improve WM performance of rats with neuropathic pain when the photo-inhibition is applied throughout the whole delay-period at shorter WM challenges (3 s), and when the photo-inhibition is applied to the late phase of the delay-period at longer WM challenges (6 s). Interestingly, both experimental groups share a performance pattern of chance level if light is applied to whole delay-period at longer WM challenges. Additionally, our findings suggest that the optogenetic stimulation of CaMKIIα-hChR2-expressing neurons in control animals decreased significantly their WM performance, but has no significant impact in the performance of pain animals.

Working-memory related neural activity was observed during the delay-period in neurons of the PFC in primates^4^ and in rodents^2^, but how chronic pain states can disrupt these activity remains unclear. Preclinical and clinical reports have suggested that chronic pain is one of the leading factors that can induce perturbations in spontaneous oscillations at different cortico-subcortical networks^37–40^, including the inhibition or facilitation of oscillatory rhythms^39,41–43^. The oscillatory interactions across different brain circuits reflect a global state of the system, and are suggested to act as an alert mechanism to identify functional perturbations^37^. In the scope of nociceptive information processing, these perturbations have been reported in the cortico-hippocampal^29,31,44^ and cortico-thalamic^30,39,45^ circuits in chronic pain patients and in animal models of chronic pain. In this study, an important aspect was the effects of bidirectional optogenetic manipulations of PL-mPFC CaMKIIα-expressing neurons activity in spectral properties. The LFP signals showed lower levels of theta frequency-band power and coherence activity during the delay-period after induction of the neuropathic pain model. These activity patterns are reversed by inhibition of PL-mPFC CaMKIIα-expressing neurons, and deteriorated by their activation. Moreover, our data showed a clear relationship between theta oscillatory activity and the critical phases of the executive demand during the WM task performance. In fact, these strong theta rhythms are believed to be necessary to the coordination of spatial mnemonic information^5,7,19,46–49^. The robust hipocampal theta oscillations has also been reported to be responsible for theta phase-locked discharge activity patterns in PL-mPFC neurons^6,7,50^. This activity has been demonstrated to be positively correlated with the behavioral performance level^6^. In agreement with these observations, we found that an enhancement of prefrontal-hippocampal theta rhythms coherence and phase-coherence during DNMS task delay-period are associated to higher WM performance levels.

To further study the prefrontal-hippocampal interplay during the cognitive performance, we computed the information flow dynamics in this circuit using a PDC analysis^34,51^. The present data showed that a clear disruption of prefrontal-hippocampal theta connectivity occurs after induction of peripheral neuropathic pain model. This marker is strongly correlated to a decline of the behavioral performance particularly in SNI-treated rats, and can be reverted when photo-inhibition is applied during the late phase of the delay-period. In this context, the behavioral trial by trial analysis showed also that correct trials are characterized by a clear increase of theta connectivity before the free-choice lever press, whereas incorrect trials are preceded by a lower connectivity level. In fact, increase of theta synchrony during the pre-stimulus phase has been previously associated to an optimization of the information shared between the hippocampus and mPFC^52^, in order to guide future correct goal-directed choices^53^.

A final note should be given on the recent insights highlighting the prefrontal cortex role in pain perception using cell-specific optogenetic for neuromodulation. It is widely supported by previous reports showing that chronic pain conditions can lead to a reorganization and deactivation of the local prefrontal cortex networks^54,55^. In the literature we can found several attempts to enhance prefrontal outputs in order to inhibit pain responses^55–60^. It has been described that Parvalbumin-containing interneurons optogenetic stimulation on the anterior cingulate cortex reduced significantly inflammatory pain sensation^61^. Another report showed a pain-related hyperactivity of prelimbic parvalbumin-expressing GABAergic interneurons, and that the activation or inhibition of these interneurons respectively increase or decrease pain responses^59^. More recently, it has been given more attention to the role of cortico-striatal interactions in pain processing. A key output target for the prefrontal cortex is the nucleus accumbens (NAc), an important component of the reward circuitry^62^. A recent report showed that activation of prefrontal projections to NAc core region relieves the sensory and affective symptoms associated with chronic pain^58^. On the other hand, the pharmacological temporary inactivation of NAc shell region with lidocaine or dopamine agonist diminishes tactile allodynia in SNI model of neuropathic pain^63,64^. In fact, the NAc shell region has been associated to an amplification of neuropathic pain responses. This increase of excitability occurs indirectly via spiny projection neurons and their inhibition can reverse injury-induced tactile allodynia^65^. Cheriyan and colleagues (2018) have also reported an altered excitability between mPFC and periaqueductal gray neurons, which may contribute to the development of the neuropathic pain phenotype^43^. Notably, in this study, we found that using our light delivery protocol, either selective inhibition or activation of PL-mPFC CaMKIIα-expressing neurons did not show any significant antinociceptive effect. The absence of changes in pain responses can be associated to the light intensity of stimulation applied. However, it is important to emphasize that our main goal was to modulate the pain-related WM performance. In fact, this dichotomy is a complex problem by the difficulty to induce the correct compensatory network balance to inhibit pain responses without interfering with cognitive component. Despite these concerns, little is known about the electrophysiological characters of prefrontal local networks and their output to other brain regions during neuropathic pain conditions. This work highlights the benefits of cell-type-targeted optogenetic manipulations to investigate the behavioral functions of specific neural subpopulations.

In summary, the present study provides new insights on how the PL-mPFC CaMKIIα-expressing neurons play an important role in the complex balance necessary to sustain prefrontal-hippocampal network inhibitory/excitatory stability and input selectivity for the processing of WM information during critical points of the cognitive demand. Together, our findings suggest that restoring the balance of PL-mPFC local network activity may be a strategy to reverse cognitive deficits observed in chronic pain conditions.

## Materials and Methods

### Animals and ethical statement

Experiments were performed in 19 Sprague-Dawley male rats (weight 275–325 g; CharlesRiver Laboratories, France). Before surgery, rats were housed in collective standard cages (type H, 3 per box), containing environment enrichment, and kept on a 12-h light/dark cycle (lights on 8 A.M.) with controlled temperature (21 ± 1 °C) and humidity (50 ± 5%). Behavioral sessions were performed at approximately the same time each day during the light portion of the circadian cycle. During the experiments, all rats were food deprived to 90–95% of their *ad libitum* feeding body weights. Rats weights were checked daily after surgery and their growth monitored weekly against a standard curve for Sprague-Dawley rats. Rats were habituated to handling by the experimenters before the start of any experimental procedures. The studies were performed in accordance with European Union (2010/63/CE) legislation and Research and Ethical Issues of the International Association for the Study of Pain^66^. The experimental protocols were also approved by the local Ethical Committee of FMUP (Porto, Portugal) and national DGAV board (Lisbon, Portugal). In this study, all efforts were made to reduce the number of animals used and to minimize their suffering.

### Surgical procedures

#### Stereotaxic intracranial virus injection and optrode implantation

The procedure for the implantation of intracranial optrodes, consisting of two multi-electrode arrays for neurophysiological recording combined with an optical fiber for optogenetic light delivery, followed the protocol described previously in detail^29,33^. The following stereotaxic coordinates (in millimeters) relative to bregma point were used to virus injection in PL-mPFC, and to place optrode structure in PL-mPFC (2.7–3.2 mm anterior to bregma, 0.5–0.9 mm lateral to midline, depth −3.4 mm), and in dCA1 (3.0–3.3 mm posterior to bregma, 2.2–2.4 lateral to midline, depth −2.7 mm)^67^. Optrodes were implanted unilaterally, and counterbalanced between left and right brain hemispheres across rats. Rats were allowed to recover for 7 days before behavioral testing sessions began.

#### Spared nerve injury

Immediately after the optrode implantation, each rat was subjected to the spared nerve injury (SNI) - model of neuropathic pain^68^ (ahead referred as SNI group, *n* = 9) or to a sham intervention (control sham group, *n* = 10) involving the same extent of skin incision and muscle dissection^68^. Both interventions were done on the hindpaw contralateral to the side of implantation of the optrode. Evaluation of sensory threshold for noxious mechanical stimulation was measured 1 hour after the end of every behavioral or recording session using von Frey filaments (Somedic, Sweden)^69^. To determine the effects of optogenetic modulation, these measurements were performed without and with optogenetic stimulation^33^.

#### Optogenetics and light delivery

Adeno-associated viruses (AAVs; serotype 5) were obtained from the University of North Carolina Vector Core. Viral titer was 6.0 × 10^12^ particles/ml for AAV5-CaMKIIα-eNpHR3.0-eYFP (sham group, *n* = 5; and SNI group, *n* = 5), and 5.9 × 10^12^ particles/ml for AAV5-CaMKIIα-hChR2(H134R)-mCherry (sham group, *n* = 5; and SNI group, *n* = 4). The use of a CaMKIIα-promoter enables transgene expression favoring excitatory pyramidal neurons^33,70^. Rats expressing inhibitory halorhodopsin (HR, eNpHR3.0 variant) in PL-mPFC were unilaterally illuminated using an orange light LED (620 nm, PlexonBright, Plexon Inc., Dallas, TX, USA) that produce a continuous solid pulse with an intensity of 5 mW at the fiber tip (irradiance = 158 mW/mm^2^), and rats expressing excitatory channelrhodopsin-2 (hChR2, H134R variant) were unilaterally illuminated using a blue light LED (465 nm) that generate pulses of 15 ms at 10 Hz with an intensity of 5 mW at the fiber tip (180 mW/mm^2^). The control experiments for non-selective potential effects of the LED light stimulation protocol in physiological and behavioral activity has been previously reported using AAV5-CaMKIIα-mCherry-expressing rats (control vector)^33^.

### Behavioral procedures

#### Apparatus and delayed non-match to sample spatial working-memory task

Behavioral performance was tested on a classical spatial WM delayed non-match to sample task (DNMS)^6^. Detailed information about the behavioral protocol applied in this study can be found in^33^. Each recording probe session was composed by 40 trials and contained an equal number of left and right rewarded levers. Optogenetic stimulation was delivered during the DNMS task delay-phase. Three different delay-period challenges were used to test behavioral performance: 1 (training phase), 3 and 6 s (testing phase). In the trials that used a delay-period of 3 s, light-delivery was applied throughout the entire delay-period; in the trials with delay-period of 6 s, light-delivery was applied throughout 2 different conditions: during the final 3 s (late phase of delay-period), or during the entire 6 s of the delay-period (whole delay-period)^33^. These light stimulation protocols were tested in separate experimental sessions. Rats were tested randomly across DNMS challenges and stimulation protocols. The experimenter was blind to group treatment. A schematic timeline on the behavioral experiments and experimental design is illustrated in Fig. 1H. Only animals that reached 90% of correct trials in the last three sessions of the learning curve (Fig. 1I) were selected to continue the testing protocol.

#### Electrophysiological recordings

Local field potentials (LFPs) recordings were obtained from implanted multielectrode arrays during behavioral DNMS task performance. The 16-channel multielectrode array was connected to a wireless head-stage transmitter (W16; Triangle Biosystems, Durham, NC, USA) that sent continuous analog signals to a Multineuron Acquisition Processor system (16-MAP, Plexon Inc). Extracellular LFPs signals were obtained by low-frequency (0.3–200 Hz) filtering of the raw signals. LFPs were preamplified and digitized at 1 kHz sampling rate. The CinePlex 2 video-tracking system (Plexon Inc) was used to provide information about the rat navigation and behavioral events on the maze and to synchronize the video recordings with the acquired neural data. Sorted files were then processed in NeuroExplorer software (NEX 4, Plexon Inc) to extract relevant behavioral and stimulation event markers. These data were subsequently analyzed in Matlab (MathWorks, Natick, MA, USA).

#### Anatomical and histological validation

After last recording session, rats were anesthetized with pentobarbital sodium (60 mg/kg, i. p.) and transcardially perfused with 0.01 M phosphate buffer, pH 7.2, in a 0.9% (w/v) saline solution, followed by 4% (v/v) paraformaldehyde. Brains were removed and post fixed in 4% paraformaldehyde for 4 h and stored in 30% (w/v) sucrose solution before they were frozen, and sectioned into 40 µm coronal slices. Brain slices were used after to identify recording and optical fibers position, and to verify tissue opsins expression. Optrode location coordinates were identified using a rat brain atlas^67^. Only rats with correct location of implanted optrode structure in the PL-mPFC and dCA1 regions, as well correct viral expression located exclusively in prelimbic area were included in the data analysis.

### Data analyses and representation

Six frequency bands of the LFPs oscillations were considered, as follows: 1–4 Hz (delta, δ), 4–9 Hz (theta, θ), 9–15 Hz (alpha, α), 15–30 Hz (beta, β), 30–50 Hz (slow-gamma, sγ), and 50–100 Hz (fast-gamma, fγ). To determine spectral power characteristics of PL-mPFC and dCA1 LFP signals, we filtered the LFP signals during DNMS delay-period in theta frequency-band using a 0-phase forward/reverse digital 4-pole Butterworth bandpass-filter^30^. Next, to determine the spectral coupling among LFP signals, we calculated the magnitude squared coherence and the phase-coherence for theta frequency-band^29,71^. To quantify the frequency-domain connectivity between PL-mPFC and dCA1 during cognitive demand, we used the statistical method of Partial directed coherence (PDC). The PDC method has been described in detail elsewhere^34,51^. The bidirectional prefrontal-hippocampal PDC activity was calculated during the DNMS delay-period and presented in respect to frequency-bands. To determine if the bidirectional PDC activity is more coactive during decision-making process around free-choice lever press, we computed the PDC activity in the period before and after lever press. We used a frequency-time function computed between 4 and 9 Hz (resolution = 1 Hz step), and between −2 and 3 s centered at lever press (resolution step, 500 ms). The PDC activity was calculated trial by trial, and represented for all trials or separated between correct and incorrect trials. In addition, to develop a behavioral response model based on the averaged bidirectional PDC activity, we calculated for each trial the averaged theta frequency-band PDC activity difference (PDC_d_) between the period after and before DNMS free-choice lever press. From all performed trials we have randomly selected 200 trials to compute a polynomial second order function (quadratic order), as a fitting activity prediction model. Using this model, we classified the performed trials as correct trials if PDC_d_ < 0, otherwise, as incorrect trials if PDC_d_ ≥ 0. Finally, we tested the efficacy of this model in predicting the occurrence of a correct or incorrect trial based only in the difference of transient connectivity difference around the free-choice lever press.

All datasets were tested for normality (GraphPad Prism 7.0, San Diego, CA, USA). The Mann-Whitney test (MW) was used to perform single comparisons, and the Kruskal-Wallis test (KW) (followed by *post hoc* Dunn’s test) or the two-factor ANOVA [F1: experimental groups × F2: frequency bands] (followed by *post hoc* Bonferroni test) to perform multiple comparisons. All effects presented as statistically significant exceeded an α-threshold of 0.05. All independence tests were two-tailed. Results are expressed as mean ± SEM.
